# Biomonitoring of metal contamination in estuarine ecosystem using seagrass

**DOI:** 10.1186/s40201-015-0198-7

**Published:** 2015-05-12

**Authors:** Faridahanim Ahmad, Shamila Azman, Mohd Ismid Mohd Said, Lavania Baloo

**Affiliations:** Department of Environmental Engineering, Faculty of Civil Engineering, Universiti Teknologi Malaysia, Johor, Malaysia

**Keywords:** Seagrass, Metal contamination, Biomonitoring, Phytoremediation

## Abstract

Metals concentrations (As, Cd, Cu, Hg and Pb) in seawater, sediment and the seagrass (*Enhalus acoroides*) were analysed at Pulai River estuary, Johor Straits, Malaysia. In this research, *Enhalus acoroides* was used in order to find it’s efficiency in up taking metals with a role in phytoremediation. Seawater, sediment and *Enhalus acoroides* samples were collected, and data of Pearson’s correlation coefficients were analysed using SPSS 16 software. Results show that lead levels were the highest metal content in *Enhalus acoroides* (202 ± 102 μg/gDW), seawater (268 ± 190 μg/L) and sediment (248 ± 218 μg/gDW), compared to other metals. There was a positive correlation for metal concentrations between *Enhalus acoroides* and sediment, but no correlation was found between *Enhalus acoroides* with seawater at estuarine area may be caused by inconsistent metal concentrations in seawater due to the influences of tidal changes and stormy waves. This indicates that *Enhalus acoroides* is a species possessing the capabilities to uptake metals from sediment, and suitable to act as both a phytoremediator and biomonitor in estuarine ecosystems due to sharp sensitivity to variation in the environment.

## Introduction

An estuary is where land meets ocean; it is known as the most valuable aquatic ecosystem where seagrass beds housing extensive marine biodiversity are located [1–4]. Aside from biodiversity, economic growth can be generated in the vicinity of estuaries i.e. the development of ports, petrochemical hubs, cities and residential areas [5, 6]. Due to increasing demand for coastal resources as well as the human population growth, the coastal ecosystem is exposed to a wider variety of pollutants; estuaries are exposed to anthropogenic contaminants including complex mixtures of heavy metals from industrial, agricultural and domestic waste, arriving via rivers or through atmospheric deposition [7–9].

Seagrass beds represent one of the most important ecological components in the coastal ecosystem; seagrass leaves act as phytoremediators and cleanse seawater by absorbing dissolved metals [10] while seagrass roots play a role in shoreline protection by reducing coastal erosion from raging storms. Indeed, the estimated value of this seagrass ecosystem conservation service amounts to a staggering US$ 34, 000 per hectare per year [11]. Moreover, seagrass can be used as a form of biomonitor to reflect the overall health of coastal waters due to their sharp sensitivity to variation in the environment [12–14]. Their abilities in phytoremediation, shoreline protection, and biomonitoring have piqued the interest of many researchers, but little supporting evidence from tropical seagrasses were ever recorded. As such, this study aims to determine the capabilities of the seagrass *Enhalus acoroides* to absorb dissolved metals from the environment (seawater and sediment) and to determine if it is suitable as a tool for biomonitoring.

## Materials and methods

### Location and sampling

**Fig. 1:**
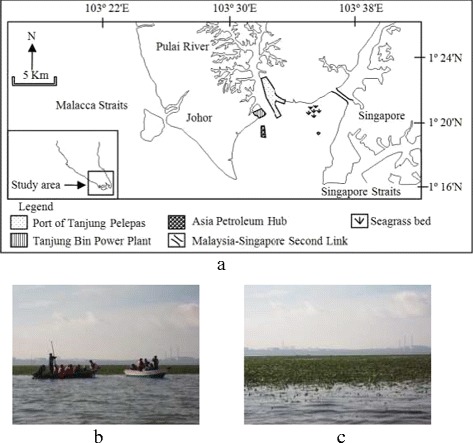
**a)** Map of location. **b)** Sampling at Pulai river estuary seagrass bed. **c)** Front view of seagrass bed

Samples of *Enhalus acoroides*, seawater and sediment were collected between July, 2011 and May, 2012 at Pulai river estuary seagrass bed, an area receiving water from Pulai River and Malacca Straits, Malaysia (Fig. 1a) [15]. The seagrass bed is located near on-going projects under the Iskandar Regional Development Authority such as the Port of Tanjung Pelepas, Tanjung Bin Power Plant and Asia Petroleum Hub [5]. Tidal of seawater at the seagrass bed was different each time and can reach low tide and high tide condition. Samples were taken during low tide condition in which the seawater level was 0–0.6 m for 1–2 h during low tide condition, the seagrass bed can appear similar to a football field as in Fig. 1b and c.

Samples of *Enhalus acoroides* were collected complete with roots, rhizomes and leaves. The samples were later rinsed with seawater to remove sediment attached to the roots and rhizomes, packed in clean plastic bags and sealed. Sediment samples were collected using a scoop and kept in a clean polyethylene container. Seawater samples were collected and kept in clean polyethylene bottles. All the packaged samples were then stored in ice en route to the laboratory and frozen at−20 °C prior to analysis.

### Sample processing and data analysis

All samples were defrosted prior to further processing. *Enhalus acoroides* samples were washed again with seawater to remove epiphytes. Following the drying method, *Enhalus acoroides* and sediment samples were dried at room temperature until they have reached a constant weight [9].

The use of higher temperatures (>60 °C) were avoided to prevent interference to metal analyses. *Enhalus acoroides* samples were then grinded using agate mortar into homogenous powder. The powdered *Enhalus acoroides* and sediment were then digested. The digestion began with placing 0.3 g homogenous *Enhalus acoroides* powder or sediment into Pyrex tubes with Teflon closure and added 5 ml HNO_3_, incubated for 1 h in a water bath at 100 °C, then cooled, filtered with 0.2 μm nylon membrane and diluted in 15 ml deionised water. Seawater samples were directly filtered with 0.2 μm nylon membrane. Metal analyses of all samples (*Enhalus acoroides*, seawater and sediment) were carried out with Perkin Elmer Atomic Absorption Spectrophotometer Model AAnalyst 400. The Pearson’s correlation coefficients of metal concentrations between *Enhalus acoroides*, seawater and sediment were analysed using SPSS version 16 software.

## Results and discussion

**Fig. 2:**
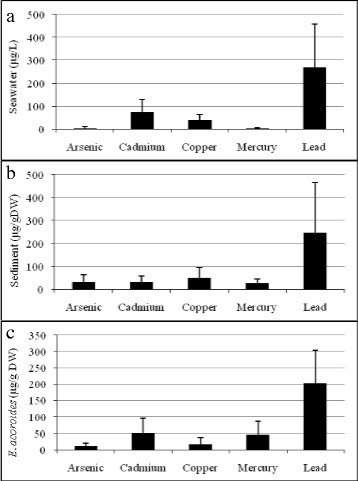
Concentrations of As, Cd, Cu, Hg and Pb on seagrass bed **a)** seawater, **b)** sediment, **c)** *Enhalus acoroides*

Figure 2a shows concentration of As, Cd, Cu, Hg and Pb in seawater. Based on Malaysian Marine Water Quality Criteria and Standard, the standard of seawater metal concentrations for class E waters (Mangroves, estuarine and river month water) was only met for As levels (3.7 ± 7.7 μg/L) however the limit were exceeded for Cd (74 ± 54 μg/L), Cu (39 ± 26 μg/L), Hg (2.7 ± 3.7 μg/L) and Pb levels (268 ± 190 μg/L) [16]. While most would assume that the port situated nearby was the root cause of the excessive metal concentration in the seawater, it is dubious since 80 % of marine pollutants usually comes from land-based activities especially the many development projects and rapid urbanisation area along the Malacca Straits [15].

All the metals that were screened and found in seawater i.e. As, Cd, Cu, Hg and Pbwere also found in sediment and *Enhalus acoroides* tissues (Fig. 2b and Fig. 2c). Pb was the highest contaminant in sediment (248 ± 218 μg/gDW) and *Enhalus acoroides* tissues (202 ± 102 μg/gDW) due to Pb being the most soluble element in water bodies from urban areas. It is not essential for plants and excessive amounts may cause growth inhabitation and even death [7, 17]. Cd concentration in *Enhalus acoroides* tissues were ranged at 52 ± 44 μg/gDW. Seagrass tissues are known to uptake Cd via physical sorption on the cell-surface and intracellular accumulation [18]. Hg concentration in *Enhalus acoroides* tissues were found to be ranged at 46 ± 41 μg/gDW. Hg released from urban areas into aquatic environments is inorganic and may convert to toxic organic methylmercury (MeHg) naturally in sediments and water columns. Once in MeHg form, they are readily uptake by seagrass [19, 20].

The range of Cu concentration in *Enhalus acoroides* tissues is 16 ± 21 μg/gDW. Cu is essential for the functioning of many proteins responsible for growth [21]. Free cupric ions (Cu^+2^) are most readily available in seawater and sediment but less in high-salinity aquatic biota [22]. As concentration in *Enhalus acoroides* tissues were in the range of 10.52 ± 10.09 μg/gDW. Unfortunately, As is a toxic element with no nutritional value for up taking [23]. The capabilities of *Enhalus acoroides* to uptake dissolved metals in similar amounts to the dissolved metals from the surroundings indicate that *Enhalus acoroides* functions well as a phytoremediator in coastal ecosystems.

In general, the factors to be considered concerning metal uptake in seagrass are the age of *Enhalus acoroides* and characteristics of surrounding seawater and sediment such as water temperature, salinity, pH, organic matter content and the presence of other metals [17]. The toxicity of marine plants that uptake metals from the surrounding (seawater and sediment) are dependent on the total concentration of up taken metals and are considered toxic at higher concentration [24, 25]. As we know, equipment used to measure the intensity of pollution in seawater and sediment is excessively expensive and requires a certain level of proficiency to operate. Observation of the presence of seagrass in coastal waters is a free and practical way to monitor pollution. In fact, it is so simple that educating the local community (fisherman and villagers) to be watchful on the degree of pollution would be an easy task.

Table 1 shows the correlation analysis of different metals between *Enhalus acoroides* with seawater and with sediment. The correlation coefficient is r, n is the number of samples, p-value is significance level settled at 0.05. There is a positive correlation for metal concentrations between *Enhalus acoroides* and sediment because roots of *Enhalus acoroides* were attach to the sediment in a longer period time and it gives time to assimilate metals consistently. Metal content at the lower part of seawater near the sediment are higher due to metal precipitation within seawater but has yet to settle onto the sediment surface [26–28]. Meanwhile, no correlations for Ar, Cd, Hg and Pb were found between *Enhalus acoroides* with seawater and a negative correlation for Cu. Such may be caused by inconsistent metal concentrations in seawater due to the influences of tidal changes (low tide and high tide) [29]. Tidal at Pulai estuary area varies from time to time, these influences inconsistent metal concentrations in seawater to be uptake by *Enhalus acoroides*, where as roots of *Enhalus acoroides* attached to the sediment as long as *Enhalus acoroides* lives and gives a longer period to assimilate metals from sediment in consistently.

**Table 1:** Results of correlation analysis between *Enhalus acoroides* with seawater and sediment

Metal	*Enhalus acoroides*-seawater			*Enhalus acoroides*-sediment		
	n	r	p-value	n	r	p-value
As	50	0	0.97	54	0.98	0.02
Cd	60	0	0.99	40	0.98	0.00
Cu	54	−0.9	0.09	49	0.98	0.02
Hg	60	0.1	0.82	54	0.86	0.14
Pb	50	0.1	0.87	54	0.45	0.55

## Conclusions

Marine pollution comes from land-based activities. After sources of metal were disposed into the river, they flow downstream and subsequently into coastal ecosystems, contributing to the dissolved metals (As, Cd, Cu, Hg and Pb) found in seawater, sediment and *Enhalus acoroides*. From this study, it was found that the metal content of *Enhalus acoroides* mirrored the metal content in sediment. There was also a positive correlation in *Enhalus acoroides* metal uptake and sediment metal content. This suggests that *Enhalus acoroides*is a species possessing the capabilities to uptake metals from sediment, and suitable to act as both a phytoremediator and biomonitor in estuarine ecosystems.
